# Towards retrieving the Promethean treasure: a first molecular assessment of the freshwater fish diversity of Georgia

**DOI:** 10.3897/BDJ.8.e57862

**Published:** 2020-10-23

**Authors:** Giorgi Epitashvili, Matthias Geiger, Jonas J Astrin, Fabian Herder, Bella Japoshvili, Levan Mumladze

**Affiliations:** 1 Institute of Zoology, Ilia State University, Tbilisi, Georgia Institute of Zoology, Ilia State University Tbilisi Georgia; 2 Zoological Research Museum A. Koenig, Bonn, Germany Zoological Research Museum A. Koenig Bonn Germany

**Keywords:** DNA barcoding, COI, molecular assessment, Caucasus

## Abstract

In this study, we provide a first estimation of the molecular diversity of the freshwater fishes of Georgia. In addition to field collections, we integrated DNA barcode data obtained from recent works and public databases (BOLD and NCBI GenBank). Currently, the DNA barcode reference library for freshwater fishes of Georgia comprises 352 DNA barcodes for 50 species, 36 genera and 15 families (52% of total Georgian freshwater fish diversity), from which 162 DNA barcodes belonging to 41 species were newly generated as part of this study. A total of 22 species are reported from the Caspian Sea basin and 31 from the Black Sea basin. Amongst the studied taxa, seven species were found with large interspecific divergences (> 2%) while 11 species were found to share DNA barcodes within our dataset. In the course of the study, we found the first evidence of the existence of *Gymnocephalus cernua* (Linnaeus, 1758) and also confirm the second occurrence of invasive *Rhinogobius lindbergi* (Berg, 1933) in Georgia. Based on the evaluation of currently-available barcode data for Georgian fishes, we highlighted major gaps and research needs to further progress DNA-based biodiversity studies in Georgia. Though this study lays a solid base for DNA, based biodiversity assessment and monitoring approaches, further efforts within the recently started CaBOL (Caucasus Barcode Of Life) project are needed to obtain reference data for the species still lacking DNA barcodes.

## Introduction

Around 15000 freshwater fish species are known worldwide (Tedesco et al. 2017). Unfortunately, many species are under threat of extinction due to heavy anthropogenic pressures, such as over-exploitation, pollution, habitat degradation and loss or invasive species (Dudgeon et al. 2007, Collen et al. 2013). Therefore, monitoring of freshwater ecosystems and their species is important for conservation planning (Dudgeon et al. 2007). The Caucasus region (and Georgia as a part thereof) is a biodiversity hotspot (Myers et al. 2000) and characterised by high diversity of landscape types and ecosystems, as well as high levels of endemism approaching up to 90% in some taxa (e.g. gastropods (Walther et al. 2014, Grego et al. 2020), millipedes (Kokhia and Golovatch 2018) etc). This is due to long-term and uninterrupted development of biodiversity in the Great and Lesser Caucasus, parts of which are suggested as plio-pleisotecene refugial areas (thus Promethean treasure) (Tarkhnishvili et al. 2012). At the same time, diversity of freshwater organisms in the Caucasus region remains poorly studied, including fishes and their distribution (Kuljanishvili et al. 2020, Mumladze et al. 2020). Unfortunately, poaching and habitat destruction (in situ gravel mining, hydropower plant constructions) threaten the endangered biota with extinction (Kuljanishvili et al. 2020, Freyhof et al. 2015). The situation is even more critical as some habitats in Georgia, such as the Rioni River, are acting as the world’s last refuge and spawning areas for critically-endangered sturgeons (*Acipenser sturio*, *A. gueldenstaedtii*, *A. nudiventri*, *A. persicus*, *A. stellatus* and *Huso huso*) (Bacalbaa-Dobrovici and Holčík 2000, Guchmanidze 2012, Kuljanishvili et al. 2020).

**Figure 1.**
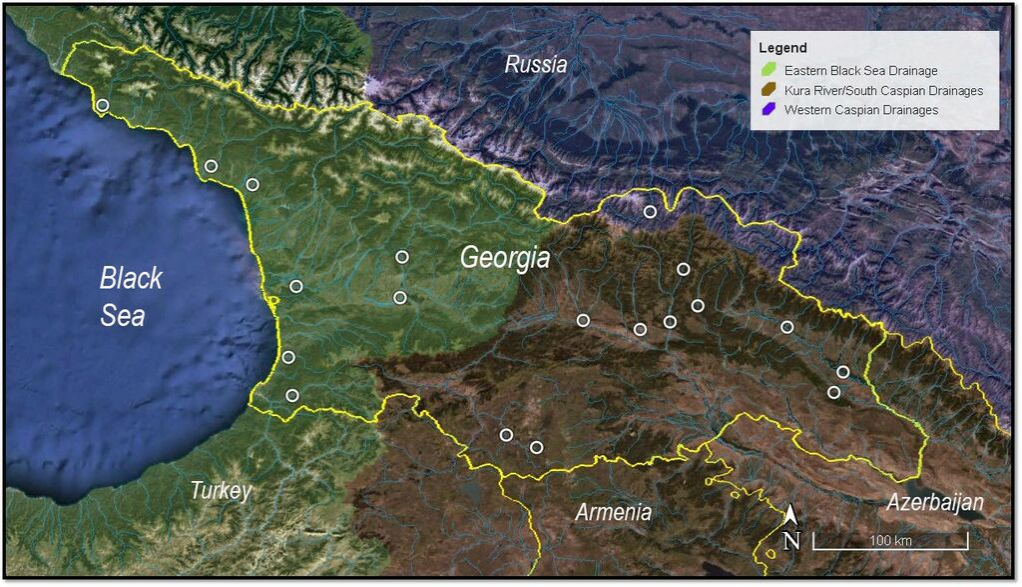
Map of Georgia (based on Google Earth Pro 7.3), showing main watersheds and river basins (delimited by shaded colours) and annotated with sampling localities (white circles).

Broadly, the Georgian ichthyofauna can be divided into the eastern Black Sea and Caspian Sea basins. According to Abell et al. (2008), Naseka (2010), within the political borders of Georgia, however, three biogeographic regions exist: A) Black Sea basin, which is separated from east Georgia by the Likhi and Meskheti ridges and covering the whole territory of west Georgia; B) Kura basin and C) Terek basin in the northern part of the country, which is separated from the Kura basin by the Greater Caucasus Mountain Range (Fig. 1).

Since the late eighteenth century, industrial and economic developments have led to severe environmental changes in the whole Caucasus region (Davtyan 2014, Freyhof et al. 2015), similar to other parts of the world (Smith et al. 1999). Since freshwater ecosystems are particularly sensitive and vulnerable to alterations in (gravel mining, hydropower plants) and adjacent to (land use) the water body (Allan and Flecker 1993), negative impacts on the ichthyofauna have likely occurred, but have never been scientifically evaluated. This is also due to a general data deficiency concerning the whole freshwater realm of the region (Mumladze et al. 2020). Along with taxonomic uncertainties in some species, major gaps still exist in the accurate knowledge of species’ distribution ranges within the Georgian inland waters, while no local conservation assessments exist for any freshwater fish species (Kuljanishvili et al. 2020). The highly-threatened group of sturgeons (*Acipenser* spp.), for example, have never been officially assessed in Georgia and only the international IUCN status is known. The Red List data for Georgian fishes are inherited from the Soviet time and thus outdated in many aspects. A new regional assessment, based on the IUCN system, is not yet available. Thus, the extent and magnitude of past disturbance or ongoing threats (such as habitat degradation, poaching, pollution and invasive species) to Georgian freshwater fishes remains largely unknown.

Along with traditional faunistic assessments, molecular genetic tools (such as DNA barcoding) have emerged as an important aid to deal with uncertainties related to taxonomy, species boundaries or cryptic diversity and have helped to enable innovative and efficient ways of biomonitoring (Hebert et al. 2003, [Bibr B6013060][Bibr B6012672], Leese et al. 2018). DNA barcoding is a method to identify species via short, standardised and easily-obtainable DNA fragments (Hebert et al. 2003, Bhattacharya et al. 2015, Lakra et al. 2015, Bingpeng et al. 2018). This method is not only limited to identifying specimens, but can also help to screen unrecognised species diversity (Barman et al. 2018).

The important step for DNA barcoding to be useful in biodiversity study/monitoring, is to develop a DNA barcode library for a particular taxa or area. The successful completion of this step, however, requires the integration of traditional taxonomic expert knowledge and DNA technology. While traditional taxonomic expertise (not only in ichthyology), based on academic training, has been largerly neglected in Georgia, as well as in many other countries — part of the phenomenon known as the ‘taxonomic impediment’ (e.g. Giangrande 2003) — DNA-based technologies for biodiversity inventories and research are gathering momentum in Georgia and can still provide new insights into freshwater fish diversity (Japoshvili et al. 2013, Ninua et al. 2018, Levin et al. 2018, Levin et al. 2019, Japoshvili et al. 2020). As a result, new, yet not fully exploited DNA-based information on Georgian fish diversity and their distribution has accumulated in recent years. Therefore, the aim of our work was to contribute to the development of a DNA barcode reference library for Georgian freshwater fishes and to summarise the current state of knowledge. We thus establish the starting point for DNA-based biodiversity evaluation and monitoring efforts of freshwater fishes of the Southern Caucasus region. The presented data will further aid in identifying taxonomically-interesting cases that need to be solved in the future.

## Materials and methods

### Data collection and DNA barcoding

In July 2018 and July 2019, concerted collecting activities (BioBlitzes) were organised by the Ilia State University - ISU (Georgia) and the Zoologisches Forschungsmuseum Alexander Koenig - ZFMK (Germany) in the Kintrishi areas in Western Georgia (N41.76 E42.02) and in the Kazbegi region in Northern Georgia (N42.65 E44.64), respectively (Thormann et al. 2019). During these events, fish sampling campaigns (permissions: #5615/01, #21/824 and #3875 – 2018/2019 issued by Ministry of Environmental Protection and Agriculture of Georgia) have been conducted in the Kintrishi and Terek River basins via electrofishing (device EFGI-650, http://www.electric-fishing.de) and frame net. After anaesthesia with MS-222 of a subsample of the collected fishes, a fin-clip was taken and stored in 99.9% molecular grade ethanol and specimens were fixed in 5-7% formaldehyde or, alternatively, specimens were directly fixed in 99% molecular grade ethanol. In addition, material collected in different areas (Fig. 1) prior to the above-mentioned activities was included (see Suppl. material 1). All specimens were identified to species level using standard morphological characters (e.g. Kottelat and Freyhof (2007)) and tissue samples of selected specimens submitted to DNA barcoding routines at ZFMK.

Genomic DNA was extracted from sub-samples using a BioSprint96 magnetic bead extractor (Qiagen, Hilden, Germany). PCRs, targeting the standard DNA barcode region COI, were carried out in 20 μl reaction volumes including 2 μl undiluted DNA template, 0.8 μl of each primer (10 pmol/μl; LCO1490-JJ: 5´-CHACWAAYCATAAAGATATYGG-3´ and HCO2198-JJ: 5´ AWACTTCVGGRTGVCCAAARAATCA-3´, (Astrin and Stüben 2008)), 2 μl ‘Q-Solution’ and 10 μl ‘Multiplex PCR Master Mix’ (Qiagen, Hilden, Germany). Thermal cycling was performed on GeneAmp PCR System 2700 machines (Applied Biosystems, Foster City, CA, USA) as follows: hot start Taq activation: 15 min at 95°C; first cycle set (15 repeats): 35 s denaturation at 94°C, 90 s annealing at 55°C (−0.2°C/cycle, ‘touch down’) and 90 s extension at 72°C. Second cycle set (25 repeats): 35 s denaturation at 94°C, 90 s annealing at 50°C and 90 s extension at 72°C; final elongation 10 min at 72°C. Purification of PCR products and bidirectional sequencing was conducted at either BGI (Hong Kong, China) or Macrogen Europe Laboratories using the amplification primers. Voucher specimens are kept in the Ichthyological collections at ISU and ZFMK. Extracted genomic DNA is deposited in the ZFMK Biobank.

### Data processing

Data processing and sequence assembly was done with the software Geneious Pro v.7 (Drummond et al. 2011) and the Muscle algorithm (Edgar 2004) was used to align the DNA barcodes after manually screening for indels or stop codons. All newly-generated DNA sequences with acceptable quality (with less than 1% ambiguous bases and free of stop codons) were submitted to the Barcode of Life Datasystem (BOLD, http://v4.boldsystems.org/), including relevant metadata where they were automatically assigned Barcode Index Numbers (BINs). They can be accessed via the public dataset “Georgian Freshwater Fishes” (DS-GGBCPIS).

In addition to the newly-generated DNA barcodes, we included all BOLD-deposited DNA barcodes that originated from Georgia. Sequences from the BOLD database were included in our dataset if the specimen metadata explicitly stated the origin of the sample and provided geo-referenced data (Suppl. material 1). Subsequently, the BOLD v4 tools evaluated sequence divergence and relationships between and within taxa, based on uncorrected *p*-distance. A Neighbour-Joining tree (based on K2P distances) with 1000 bootstrap replicates was constructed to investigate congruence between morphological identity and genetic relationships. Analyses were performed using MEGA X software (Kumar et al. 2018) and statistical tools provided by BOLD Systems (Ratnasingham and Hebert 2007).

## Results and discussion

DNA barcodes of 352 individuals representing 50 species, 36 genera and 15 families (52% of Georgian freshwater fish diversity) are currently available for Georgian fishes. From these, 162 COI sequences were newly generated for this study, through the GGBC (Georgian-German Biodiversity Center) initiative, 153 were contributed through the FREDIE (Freshwater Diversity Distribution for Europe) project (https://www.fredie.eu/), 19 sequences stem from the "Russian Freshwater Fishes" project on BOLD and 18 DNA barcodes were mined from GenBank through BOLD. In the final dataset of all 352 barcodes, the length of the COI sequences was, on average, 648 base pairs (minimum 465 and maximum 658) including no stop codons, insertions or deletions. A total of 82 positions out of 658 (13%) were variable, from which 60 positions (9%) were diagnostically informative. On average, nucleotide base frequency (A-24.47%, C-27.67%, G-18.56%, T-29.29%) and GC content (46.24%) were well within the range known for fishes (see, for example, Bingpeng et al. (2018)). Distance summary statistics are provided in Table 1, showing significant changes of average *p*-distance amongst family, genus and species level.

**Figure 2.**
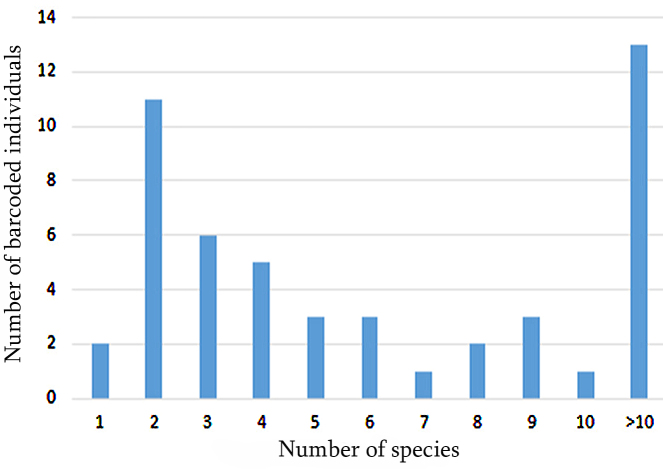
Barcode frequency distribution for Georgian fish species in a BOLD System at the time of writing.

**Figure 3.**
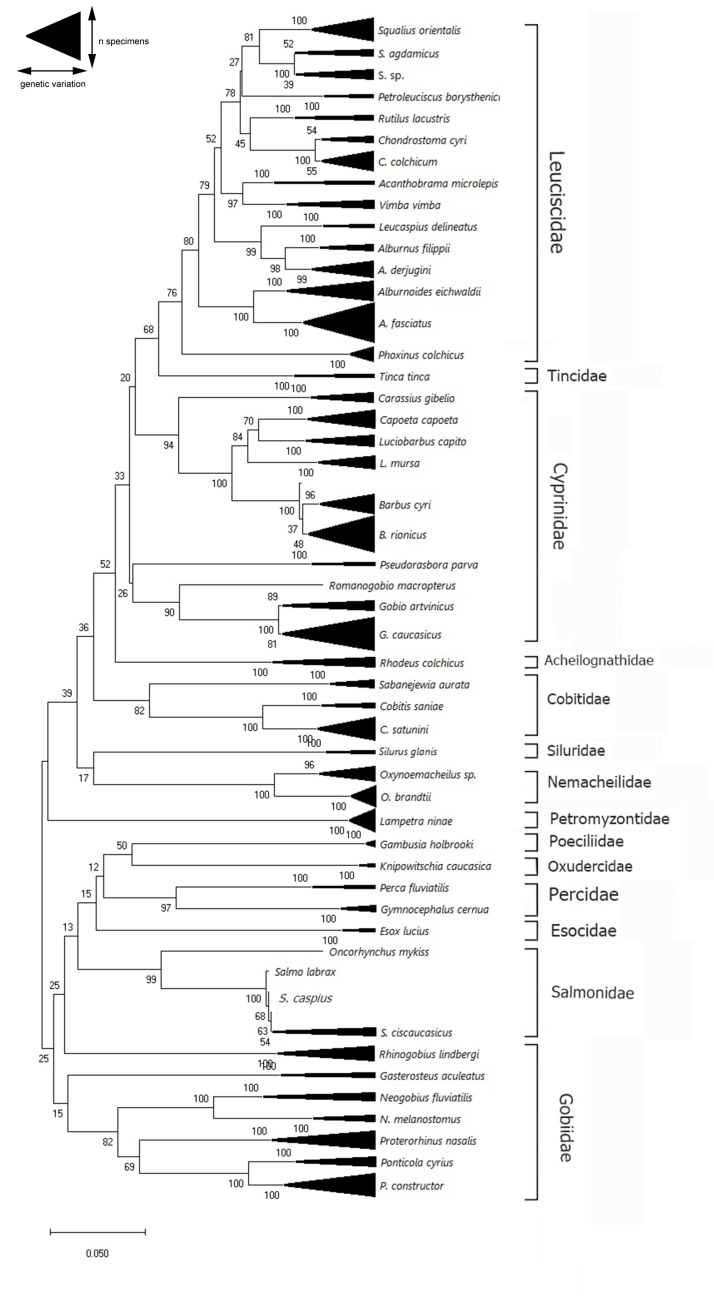
Compact Neighbour-Joining tree based on mitochondrial COI barcode region using K2P distance model with other default parameters provided by MegaX software. Numbers near nodes indicate bootstrap support values from 1000 replicates. The analyses involved all 352 COI nucleotide sequences.

Amongst the 50 barcoded species, 26 were represented by more than four sequences in the dataset allowing intraspecific distance estimates (Fig. 2; Suppl. material 2). The highest number of barcodes per genus was reached for the genus *Alburnoides* with two species: *A. fasciatus* (24 barcodes) and *A. eichwaldii* (12 barcodes), followed by the genus *Barbus* represented also by two species: *B. rionicus* (23 barcodes) and *B. cyri* (12 barcodes). Most species however, are represented by less than 10 DNA barcodes. Two species, *Romanogobio macropterus* and *Oncorhynchus mykiss* are represented by a single DNA barcode. Summarised information on barcoded specimens for each species, intraspecific distances and nearest neighbours according to the ‘Barcode Gap Analysis’ tool available in BOLD Systems are provided in Suppl. material 2. The full Neighbour-Joining tree, including all specimens (Fig. 3), resolved almost all morphological species as unique clusters in congruence with morphological species identification. However, three of the genera (*Chondrostoma*, *Gobio, Salmo*) showed complicated sequence relationships where maximum within-species distances were larger than the minimum between-species distances amongst the congenerics (Suppl. material 2), with also low bootstrap support for species level clusters on the tree (Fig. 3).

Due to small interspecific genetic distances (i.e. distance to nearest neighbour), several species were not predicted to be separate taxa by barcode gap analyses. For instance, nearest-neighbour distances for species belonging to *Salmo*, *Barbus*, *Capoeta*, *Chondrostoma*, *Gobio* and *Squalius*, showed < 2% divergence, indicating a possible need for re-evaluating species limits. As an example, all three specimens from Natanebi and Supsa rivers (Western Georgia, Black Sea Basin), which were identified as *Gobio artvinicus* (sequence IDs in BOLD: EUFWF3080-18; EUFWF3079-18; EUFWF4984-19) according to Turan et al. (2016), though are represented as a separate cluster in the COI cladogram Fig. 3, have near-zero divergence with the *G. caucasicus* (23 specimens from Kura River - Caspian Sea Basin). This might be considered as a result of geographic divergence between populations rather than attributing the populations to different species. Thus, additional integrative taxonomic investigation could help to solve the systematics of the genus *Gobio* in Georgia. Though the species of some of those genera (*Salmo*, *Barbus*, *Gobio*) were (re)evaluated as valid species relatively recently (Turan et al. 2016, Ninua et al. 2018, Aksu and Bektaş 2019, Levin et al. 2019, Kuljanishvili et al. 2020), increasing the sampling (adding both species-level taxa and barcodes per species) and geographic coverage is necessary to develop an effective barcode library.

**Figure 4.**
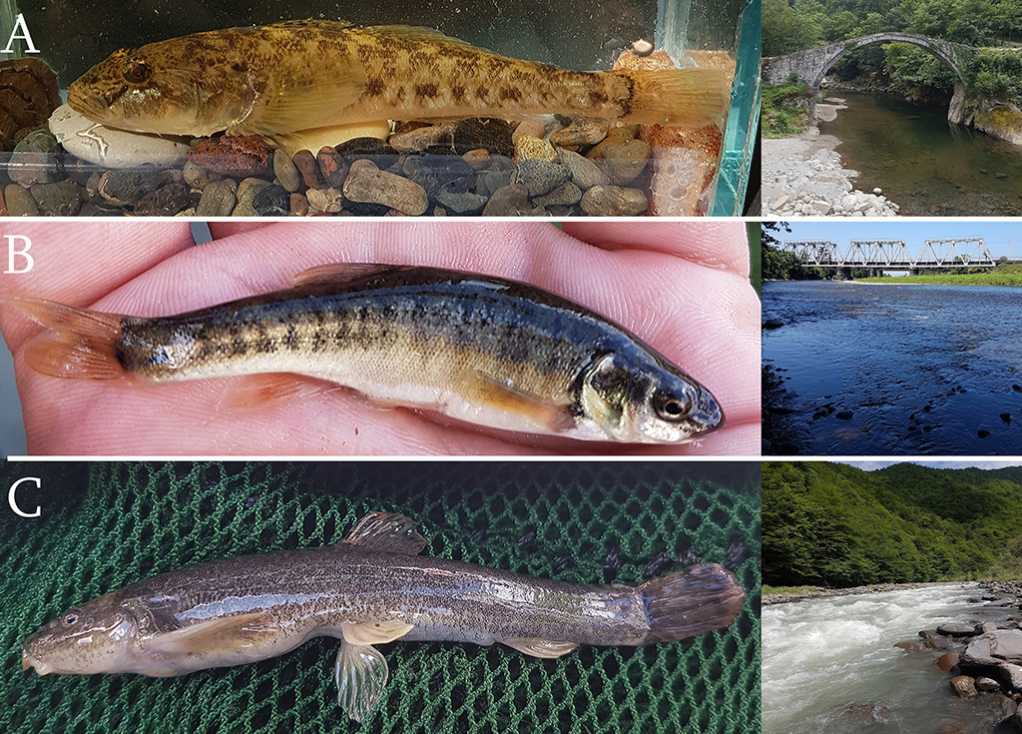
Sample of taxa studied in this article and their habitats. A - *Neogobius fluviatilis* (downstream of Kintrishi River); B - *Phoxinus colchicus* (Kintrishi River, close to river mouth); C - *Oxynoemacheilus* sp. (Lajanuri River - right tributary of Rioni river).

Seven species (Chondrostoma cyri, Rutilus lacustris, Alburnus derjugini, Phoxinus colchicus, Oxynoemacheilus sp., *Neogobius fluviatilis* and *Proterorhinus nasalis*) (Fig. 4) showed maximum interspecific divergence larger than 2% (*p*-distance) pointing them out as interesting subjects for further in-depth studies. All these species await additional studies to clarify their taxonomic positions. The genera *Chondrostoma, Rutilus, Alburnus, Neogobius* and *Oxynoemacheilus* are diverse (i.e. more than two species according to Kuljanishvili et al. (2020)) in the Caucasus region for which unambiguous systematics, as well as detailed distribution of separate species or genetic profiling, is still lacking. For instance, Kuljanishvili et al. (2020) assume the occurrence of four species of *Rutilus* in the South Caucasus. However, a study, based on the Cytochrome b marker by Levin et al. (2017), suggests the possibility that only a single genetically-highly polymorphic *R. lacustris* occurs in the Caucasus region. Likewise, the systematics of Caucasian species and genera of Gobiidae and Nemacheilidae are amongst the most confusing, as already pointed out by Kuljanishvili et al. (2020). For these taxa, our COI barcode dataset is insufficient in order to draw meaningful conclusions; however, large intraspecific distances further indicate possible yet undescribed diversity within these taxa. For instance, regionally monospecific genera with high intraspecific genetic distances, such as *Phoxinus* and *Proterorhinus*, are interesting taxa that might be represented by genetically deeply-structured populations (if not cryptic species complexes) associated with Black vs Caspian Sea basins.

**Figure 5.**
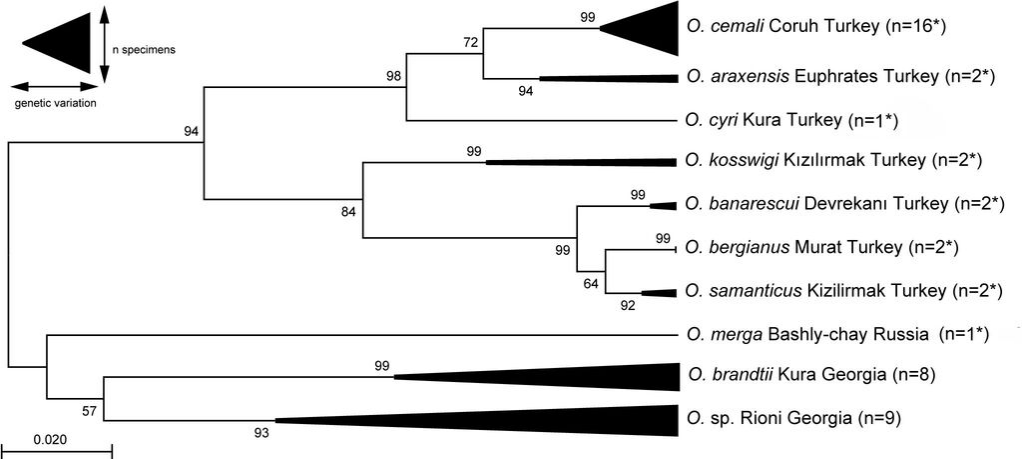
Maximum Likelihood estimation of the phylogenetic relationships of *Oxynoemacheilus* loaches, based on the mitochondrial COI barcode region (Kimura 2-parameter model, discrete Gamma distribution for rate differences with three categories + G parameter = 0.0610). Nucleotide positions with less than 95% site coverage were eliminated, resulting in 637 analysed positions. Numbers near nodes indicate bootstrap support values from 1000 pseudoreplicates. The tree is drawn to scale, with branch lengths measured in the number of substitutions per site. The analysis includes 45 nucleotide sequences taken from Turan et al. (2019) (for asterisked species, NCBI GenBank accession numbers are given in Suppl. material 3) of this study.

Loaches of the genus *Oxynoemacheilus* from the Black Sea basin have recently been studied including the description of a new species – *O. cemali* – from the Coruh river drainage (Turan et al. 2019). The Coruh enters the Black Sea near Batumi in Georgia about 70 km south of the Rioni River mouth. We therefore compared the DNA barcodes made available by Turan et al. (2019) to the newly-generated COI sequences, also to test whether there is molecular support for the Rioni loach belonging to *O. cemali*. The comparison showed that the Rioni *Oxynoemacheilus* (Fig. 4) is genetically closer to *O. brandtii* from the Kura River (7.5% min. K2P distance) than to the presumably also Georgian *O. cemali* (11.9% min. K2P distance). It is, therefore, highly unlikely that the Rioni loach belongs to one of the two species (Fig. 5). As the taxonomic status of *Oxynoemacheilus* species in the study area is only partially understood (e.g. *O. angorae alasanicus*, *O. bergi*, *O. brandti gibbusnazus* or *O. lenkoranensis* – see Kuljanishvili et al. (2020)), further studies with larger series of adult specimens are needed to address these issues and allow taxonomic sound examinations and conclusions.

DNA barcoding confirmed for the first time an occurrence of *Gymnocephalus cernua* (Linnaeus, 1758) and contributed the second record of alien *Rhinogobius lindbergi* (Berg, 1933) in Georgia. The former species has never been considered to occur in the country (Elanidze 1983, Ninua and Japoshvili 2008, Kuljanishvili et al. 2020), but is abundant in the adjacent northern Caucasus area (Kottelat and Freyhof 2007). Possibly, the species currently extends its range to the Southern Caucasus, although reports from fishermen are lacking. As the species is a strong invader and has drastically expanded its range over the last decades (e.g. to the North American Great Lakes, where it possibly poses a threat to their endemic fish fauna (Gunderson et al. 1998, Newman 1999), monitoring its status in Georgia is recommended. *Rhinogobius lindbergi* was recently first reported from Georgia as an alien species (Japoshvili et al. 2020). Our data confirm the finding and further indicates the widespread distribution of this species in eastern Georgia as already supposed by Japoshvili et al. (2020). The likely introduction pathways or vectors for this species are currently unknown. The direct migration from southern Caspian rivers is probably impossible due to impermeable barriers at the Mingachevir reservoir. Accordingly, *R. lindbergi* have been introduced in eastern Georgian rivers by humans, most probably unintentionally.

## Conclusions

Georgia, as part of the Caucasus and Irano-Anatolian biodiversity hotspots, is distinguished by its unique biodiversity and rich freshwater resources, which have been strongly impacted by anthropogenic pressure throughout the 20^th^ century until the present. During the Soviet time, large-scale industrial projects presumably had a strong influence on the Georgian biodiversity and especially on the freshwater fauna. An example of this is the construction of the Mingachevir Dam in Azerbaijan which acted as an insurmountable obstacle for anadromous fishes such as sturgeons, Caspian lampreys and salmon (Kuljanishvili et al. 2020). As a result, these species (in particular, lampreys and sturgeons) lost the spawning areas in the whole upper Kura basin. In addition, from the 1930s to 1980s, alien species were introduced intentionally or accidentally to Georgia, such as gibel carp and topmouth gudgeon (Ninua and Japoshvili 2008, Japoshvili et al. 2013, Kuljanishvili et al. 2020). Although large industrial projects were halted with the collapse of the Soviet Union, the recent extensive development of small and medium-sized hydropower plants in Georgia will presumably have negative impacts on the local freshwater biodiversity. In addition, illegal fishing, range expansion of non-native species, water pollution and habitat modification will alter the population dynamics and distribution of most native freshwater fishes of Georgia, including rare, endemic and especially anadromous species. Given these expectations, intensive study and monitoring of fishes is highly recommended to estimate population changes and species distribution and for subsequent planning of conservation activities and mitigation of irreversible diversity loss. The fastest and perhaps most cost-effective tools in this regard will be methods based on DNA barcoding (Kress et al. 2015). However, our study shows that the number of reference barcodes available for Georgian fishes is not yet sufficient to implement full-scale fish diversity monitoring programmes. Indeed, the barcodes of nearly 50% of Georgian fish species are not yet available. This is mainly due to the limited financial/human resources to investigate the fish diversity on one hand and also due to poor museum collections available for Georgian fishes. For example, the largest fish collection kept in the Georgian National Museum has been damaged so badly (as a result of incorrect preservation) that it is no longer useful for genetic study. Thus unresolved taxonomy (such as *Oxynoemacheilus* or *Squalius*) and insufficient barcode coverage are currently major gaps that need to be filled in the near future. We hope this is indeed possible, given the relatively-low species number (compared to mega-diverse regions) and the existing progress in fish research in the region. The recently-initiated CaBOL project (Caucasus Barcode of Life) constitutes a chance to close some of these taxonomic gaps over the next few years.

## Supplementary Material

EA5A5755-FC22-5210-9BB8-CEB05696839C 10.3897/BDJ.8.e57862.suppl1 Supplementary material 1 Epitashvili et al. Supplementary Table S1. Data type Occurrences, Collection data Brief description Details on barcoded specimens from Georgia currently available in BOLD. Abbreviations used in the table: NA - information is not available; ISU - Ilia State University; FJF - Fischsammlung Jorg Freyhof; PC - Prior Collection i.e. before 2018-2019 bioblitz; RAS - Russian Academy of Sciences, Zoological Institute; ZFMK - Zoologisches Forschungsmuseum Alexander Koenig. Samples in bold face are mined from GenBank with BOLD ID same as GenBank accession number. File: oo_454824.xlsx https://binary.pensoft.net/file/454824 Epitashvili G, Geiger M, Astrin J, Herder F, Japoshvili B, Mumladze L

D9B077A6-F0CB-500A-8127-64521F56616E 10.3897/BDJ.8.e57862.suppl2 Supplementary material 2 Epitashvili et al. Supplementary Table S2. Data type Summarized data Brief description Taxonomic structure and BOLD summary data of barcoded Georgian fishes with mean and maximum intraspecific distances and nearest-neighbour statistics. File: oo_444692.xlsx https://binary.pensoft.net/file/444692 Epitashvili G, Geiger M, Astrin J, Herder F, Japoshvili B, Mumladze L

190D6734-ED8C-5C95-9AAF-96926C29E784 10.3897/BDJ.8.e57862.suppl3 Supplementary material 3 GenBank accession numbers for the sequences used in Figure 5. Data type Genomic File: oo_456860.xlsx https://binary.pensoft.net/file/456860 Epitashvili G, Geiger M, Astrin J, Herder F, Japoshvili B, Mumladze L

## Figures and Tables

**Table 1.** Summary table of K2P genetic distances within the different taxonomic levels derived from 349 specimens analysed. The list of studied species is provided in Suppl. material 1

**Label**	**N**	**Taxa**	**Comparisons**	**Min Dist (%)**	**Mean Dist (%)**	**Max Dist (%)**	**S.E.**
**Within Species**	349	50	1835	0.00	0.33	2.46	0.01
**Within Genera**	253	13	1268	0.00	4.1	9.54	0.00
**Within Families**	295	5	21804	4.00	16.1	27.98	0.00

## References

[B6012417] Abell R., Thieme M. L., Revenga C., Bryer M., Kottelat M., Bogutskaya N., Coad B., Mandrak N., Balderas S. C., Bussing W., Stiassny M. L.J., Skelton P., Allen G. R., Unmack P., Naseka A., Ng R., Sindorf N., Robertson J., Armijo E., Higgins J. V., Heibel T. J., Wikramanayake E., Olson D., Lpez H. L., Reis R. E., Lundberg J. G., Sabaj Prez M. H., Petry P. (2008). Freshwater ecoregions of the World: A new map of biogeographic units for freshwater biodiversity conservation. BioScience.

[B6012450] Aksu I., Bektaş Y. (2019). Mitochondrial phylogeny and biogeography of the genus *Gobio* (Teleostei: Cyprinidae) in Turkey. Zoology in the Middle East.

[B6012459] Allan J. D., Flecker A. S. (1993). Biodiversity conservation in running waters: Identifying the major factors that threaten destruction of riverine species and ecosystems. BioScience.

[B6012468] Astrin Jonas J., Stüben Peter E. (2008). Phylogeny in cryptic weevils: molecules, morphology and new genera of western Palaearctic Cryptorhynchinae (Coleoptera:Curculionidae). Invertebrate Systematics.

[B6012477] Bacalbaa-Dobrovici N., Holčík J. (2000). Distribution of *Acipenser
sturio* L., 1758 in the Black Sea and its watershed. Boletin - Instituto Espanol de Oceanografi.

[B6012486] Barman A. S., Singh M., Singh S. K., Saha H., Singh Y. J., Laishram M., Pandey P. K. (2018). DNA barcoding of freshwater fishes of Indo-Myanmar biodiversity hotspot. Scientific Reports.

[B6012498] Bhattacharya M., Sharma A. R., Patra B. C., Sharma G., Seo E., Nam J., Chakraborty C., Lee S. (2015). DNA barcoding to fishes: current status and future directions. Mitochondrial DNA Part A.

[B6012538] Bingpeng X, Heshan L, Zhilan Z, Chunguang W, Yanguo W, Jianjun W (2018). DNA barcoding for identification of fish species in the Taiwan Strait. PLOS One.

[B6012549] Collen Ben, Whitton Felix, Dyer Ellie E., Baillie Jonathan E. M., Cumberlidge Neil, Darwall William R. T., Pollock Caroline, Richman Nadia I., Soulsby Anne-Marie, Böhm Monika (2013). Global patterns of freshwater species diversity, threat and endemism. Global Ecology and Biogeography.

[B6012564] Davtyan E. (2014). The role of infrastructure in international relations: the case of South Caucasus. International Journal of Social Sciences.

[B6012519] Drummond A, Ashton B, Buxton S, Cheung M, Cooper A, Duran C, Field M, Heled J, Kearse M, Markowitz S, Moir R, Stones-Havas S, Sturrock S, Thierer T, Wilson A (2011). GENEIOUS Pro. http://www.geneious.com.

[B6012573] Dudgeon David, Arthington Angela H., Gessner Mark O., Kawabata Zen-Ichiro, Knowler Duncan J., Lévêque Christian, Naiman Robert J., Prieur-Richard Anne-Hélène, Soto Doris, Stiassny Melanie L. J., Sullivan Caroline A. (2007). Freshwater biodiversity: importance, threats, status and conservation challenges. Biological Reviews.

[B6012589] Edgar R. C. (2004). MUSCLE: multiple sequence alignment with high accuracy and high throughput. Nucleic Acids Research.

[B6012598] Elanidze R. (1983). Ichthyofauna of the rivers and lakes of Georgia.

[B6012614] Freyhof J., Khorozyan I., Sadigov F., Japoshvili B., Batsatsashvili K., Fayvush G., Shukurov E., Beruchashvili G., Arobelidze N., Kandaryan A., Bitsadze M., Zazanashvili N., Manvelyan K., Askerov E., Karapetyan K., Ahmadova K. (2015). Towards sustainable dam and hydropower in the south Caucasus.

[B6012634] Giangrande Adriana (2003). Biodiversity, conservation, and the 'Taxonomic impediment'. Aquatic Conservation: Marine and Freshwater Ecosystems.

[B6012660] Grego Jozef, Mumladze Levan, Falniowski Andrzej, Osikowski Artur, Rysiewska Aleksandra, Palatov Dimitry M., Hofman Sebastian (2020). Revealing the stygobiotic and crenobiotic molluscan biodiversity hotspot in Caucasus: Part I. The phylogeny of stygobiotic Sadlerianinae Szarowska, 2006 (Mollusca, Gastropoda, Hydrobiidae) from Georgia with descriptions of five new genera and twenty-one new species. ZooKeys.

[B6012643] Guchmanidze A (2012). Sturgeons of Georgian Black Sea coast, their genesis, taxonomic composition, bio ecology, otolith structure and conservation.

[B6012651] Gunderson Jeffrey L., Klepinger Michael R., Bronte Charles R., Marsden J. Ellen (1998). Overview of the international symposium on Eurasian ruffe (*Gymnocephalus
cernuus*) Biology, Impacts, and Control. Journal of Great Lakes Research.

[B6012672] Hajibabaei Mehrdad (2012). The golden age of DNA metasystematics. Trends in Genetics.

[B6012681] Hebert Paul D. N., Cywinska Alina, Ball Shelley L., deWaard Jeremy R. (2003). Biological identifications through DNA barcodes. Proceedings of the Royal Society of London. Series B: Biological Sciences.

[B6012699] Japoshvili Bella, Mumladze Levan, Küçük Fahrettin (2013). Invasive Carassius carp in Georgia: Current state of knowledge and future perspectives. Current Zoology.

[B6012708] Japoshvili B., Lipinskaya T., Gajduchenko H., Sinchuk A., Bikashvili A., Mumladze L. (2020). Two new alien species records from the republic of Georgia. Acta Zoologica Bulgarica.

[B6012736] Kokhia Mzia S., Golovatch Sergei I. (2018). A checklist of the millipedes of Georgia, Caucasus (Diplopoda). ZooKeys.

[B6012754] Kottelat M, Freyhof J (2007). Handbook of European Freshwater Fishes.

[B6012762] Kress W. John, García-Robledo Carlos, Uriarte Maria, Erickson David L. (2015). DNA barcodes for ecology, evolution, and conservation. Trends in Ecology & Evolution.

[B6012771] Kuljanishvili Tatia, Epitashvili Giorgi, Freyhof Jörg, Japoshvili Bella, Kalous Lukáš, Levin Boris, Mustafayev Namig, Ibrahimov Shaig, Pipoyan Samvel, Mumladze Levan (2020). Checklist of the freshwater fishes of Armenia, Azerbaijan and Georgia. Journal of Applied Ichthyology.

[B6012786] Kumar Sudhir, Stecher Glen, Li Michael, Knyaz Christina, Tamura Koichiro (2018). MEGA X: Molecular Evolutionary Genetics Analysis across computing platforms. Molecular Biology and Evolution.

[B6012796] Lakra Wazir Singh, Singh M., Goswami Mukunda, Gopalakrishnan A., Lal K. K., Mohindra V., Sarkar U. K., Punia P. P., Singh K. V., Bhatt J. P., Ayyappan S. (2015). DNA barcoding Indian freshwater fishes. Mitochondrial DNA Part A.

[B6012812] Leese Florian, Bouchez Agnès, Abarenkov Kessy, Altermatt Florian, Borja Ángel, Bruce Kat, Ekrem Torbjørn, Čiampor Fedor, Čiamporová-Zaťovičová Zuzana, Costa Filipe O., Duarte Sofia, Elbrecht Vasco, Fontaneto Diego, Franc Alain, Geiger Matthias F., Hering Daniel, Kahlert Maria, Kalamujić Stroil Belma, Kelly Martyn, Keskin Emre, Liska Igor, Mergen Patricia, Meissner Kristian, Pawlowski Jan, Penev Lyubomir, Reyjol Yorick, Rotter Ana, Steinke Dirk, van der Wal Bas, Vitecek Simon, Zimmermann Jonas, Weigand Alexander M. (2018). Why we need sustainable networks bridging countries, disciplines, cultures and generations for aquatic biomonitoring 2.0: A perspective derived from the DNAqua-Net COST Action. Next Generation Biomonitoring: Part 1.

[B6012869] Levin Boris A., Gandlin Alexander A., Simonov Evgeniy S., Levina Marina A., Barmintseva Anna E., Japoshvili Bella, Mugue Nikolai S., Mumladze Levan, Mustafayev Namig J., Pashkov Andrey N., Roubenyan Haikaz R., Shapovalov Maxim I., Doadrio Ignacio (2019). Phylogeny, phylogeography and hybridization of Caucasian barbels of the genus *Barbus* (Actinopterygii, Cyprinidae). Molecular Phylogenetics and Evolution.

[B6012849] Levin B. A., Simonov E. P., Ermakov O. A., Levina M. A., Interesova E. A., Kovalchuk O. M., Malinina Y. A., Mamilov N. S., Mustafayev N. J., Pilin D. V., Pozdeev I. V., Prostakov N. I., Roubenyan H. R., Titov S. V., Vekhov D. A. (2017). Phylogeny and phylogeography of the roaches, genus *Rutilus* (Cyprinidae), at the Eastern part of its range as inferred from mtDNA analysis. Hydrobiologia.

[B6128988] Levin B. A., Simonov E., Matveyev M. P., Artaev O. N., Mustafayev N. J., Pashkov A. N., Roubenyan H. R. (2018). DNA barcoding of the fishes of the genus Alburnoides (Actinopterygii, Cyprinidae) from Caucasus. Mitochondrial DNA Part A.

[B6012889] Mumladze Levan, Japoshvili Bella, Anderson Elizabeth P. (2020). Faunal biodiversity research in the Republic of Georgia: a short review of trends, gaps, and needs in the Caucasus biodiversity hotspot. Biologia.

[B6012898] Myers Norman, Mittermeier Russell A., Mittermeier Cristina G., da Fonseca Gustavo A. B., Kent Jennifer (2000). Biodiversity hotspots for conservation priorities. Nature.

[B6130236] Naseka A. (2010). Zoogeographical Freshwater Divisions of the Caucasus as a part of the West Asian Transitional Region. Proceedings of the Zoological Institute RAS.

[B6012908] Newman R. M. (1999). Ruffe - a problem or just a pest?. Aquatic Nuisance Species Digest.

[B6012935] Ninua Levan, Tarkhnishvili David, Gvazava Elguja (2018). Phylogeography and taxonomic status of trout and salmon from the Ponto-Caspian drainages, with inferences on European brown trout evolution and taxonomy. Ecology and Evolution.

[B6012917] Ninua N. S., Japoshvili B. O. (2008). Check list of fishes of Georgia. Proceedings of the Institute of Zoology.

[B6012948] Ratnasingham S., Hebert P. D. (2007). BOLD: The Barcode of Life Data System (http://www.barcodinglife.org). Molecular Ecology Notes.

[B6128974] Smith V. H., Tilman G. D., Nekola J. C. (1999). Eutrophication: Impacts of excess nutrient inputs on freshwater, marine, and terrestrial ecosystems. Environmental Pollution.

[B6033744] Tarkhnishvili D., Gavashelishvili A., Mumladze L. (2012). Palaeoclimatic models help to understand current distribution of Caucasian forest species. Biological Journal of the Linnean Society.

[B6012967] Tedesco Pablo A., Beauchard Olivier, Bigorne Rémy, Blanchet Simon, Buisson Laëtitia, Conti Lorenza, Cornu Jean-François, Dias Murilo S., Grenouillet Gaël, Hugueny Bernard, Jézéquel Céline, Leprieur Fabien, Brosse Sébastien, Oberdorff Thierry (2017). A global database on freshwater fish species occurrence in drainage basins. Scientific Data.

[B6012988] Thormann J., Ahrens D., Anderson C., Astrin J. J., Mumladze L., Rulik B., Tarkhnishvili D., Espeland M., Geiger M., Hein N., Iankoshvili G. (2019). A prelude to the Caucasus Barcode of Life Platform (CaBOL): Biodiversity Days in Georgia in 2018 and 2019. Bonn Zoological Bulletin.

[B6013004] Turan Davut, Japoshvili Bella, Aksu İsmail, Bektaş Yusuf (2016). Description of two new species of the genus *Gobio* (Teleostei: Cyprinidae) from the Black Sea coast of Turkey. Zoology in the Middle East.

[B6013013] Turan Davut, Kaya Cüneyt, Kalayci Gökhan, Bayçelebi Esra, Aksu İsmail (2019). *Oxynoemacheilus
cemali*, a new species of stone loach (Teleostei: Nemacheilidae) from the Çoruh River drainage, Turkey. Journal of Fish Biology.

[B6013023] Walther F., Kijashko P., Harutyunova L., Mumladze L., Neiber M. T., Hausdorf B. (2014). Biogeography of the land snails of the Caucasus region. Tentacle.

[B6013060] Waugh John (2007). DNA barcoding in animal species: progress, potential and pitfalls. BioEssays.

